# Aglepristone Administration in Mid-Proestrus Reduces the LH Peak but Does Not Prevent Ovulation in the Bitch

**DOI:** 10.3390/ani11071922

**Published:** 2021-06-28

**Authors:** Piotr Socha, Katarzyna Bladowska, Sławomir Zduńczyk, Tomasz Janowski

**Affiliations:** Department of Animal Reproduction with Clinic, University of Warmia and Mazury, Oczapowskiego 14, 10-719 Olsztyn, Poland; kasiamalinowska1986@gmail.com (K.B.); zdun@uwm.edu.pl (S.Z.); jantom@uwm.edu.pl (T.J.)

**Keywords:** aglepristone, periovulatory phase, progesterone

## Abstract

**Simple Summary:**

The role of preovulatory progesterone for LH release and ovulation in the bitch is not clear. The aim of the study was to evaluate the influence of administration of aglepristone in mid-proestrus on progesterone concentration, LH release, and occurrence of ovulation in the bitch. Experimental bitches (*n* = 7) were treated on days 4 and 5 of proestrus with aglepristone (Alizin^®^, Virbac) at the dose of 10 mg/kg body weight s.c. (i.e., the two treatments were 24 h apart). The progesterone concentration showed a similar pattern in both groups. The LH peak value and area under the curve for LH in bitches treated with aglepristone were significantly lower than those in control bitches. The ovulation occurred in all animals in both groups. The presented study showed that withdrawal of progesterone by administration of aglepristone in the mid-proestrus significantly reduced the preovulatory LH surge, but it had no effect on periovulatory progesterone concentration or the occurrence of ovulation.

**Abstract:**

The aim of the study was to evaluate the influence of administration of aglepristone in mid-proestrus on progesterone concentration, LH release, and occurrence of ovulation in the bitch. Experimental bitches (*n* = 7) were treated on days 4 and 5 of proestrus with aglepristone at the dose of 10 mg/kg body weight s.c. (i.e., the two treatments were 24 h apart). Control animals (*n* = 7) received s.c. injections of saline. For progesterone determination, blood was collected daily until the first day of cytological diestrus. For LH determination, blood was collected daily and in the periovulatory phase every 8 h. The progesterone concentration showed a similar pattern in both groups. The LH peak value in bitches treated with aglepristone was significantly lower (*p* < 0.05) than that in control bitches (4.83 ± 1.20 vs. 13.66 ± 1.21 ng/mL). The area under the curve (AUC) for LH was significantly (*p* < 0.05) lower in treated than in control animals (6.85 ± 1.21 ng/mL/d vs. 12.25 ± 1.35 ng/mL/d). The ovulation occurred in all animals in both groups. The study showed that administration of aglepristone in the mid-proestrus significantly reduced the preovulatory LH surge, but it had no effect on progesterone concentration and the occurrence of ovulation.

## 1. Introduction

The periovulatory phase is critical in breeding management in the dog; however, relatively little is known about the regulation of the canine reproductive processes during this time [1]. Proestrus is associated with development of ovarian follicles and secretion of estradiol from the granulosa cells. During proestrus, estradiol concentrations increase from 5 to 15 pg/mL initially, reaching peak levels of 40–120 pg/mL, and then begin to decline one day before the surge of luteinizing hormone (LH) [2,3]. Progesterone (P4) concentration rises very slowly throughout proestrus, from basal values of 0.2–0.4 ng/mL to 1.5–2 ng/mL before or at the start of the LH surge [2,4]. This preovulatory P4 increase results from the luteinization of ovarian follicles, visible histologically as early as 6 days before ovulation [1,5].

The LH surge onset typically occurs 1–2 days after the peak in estradiol. LH concentration is elevated for 2 days on average, and LH peak levels range from 3 to 40 ng/mL [5,6]. Ovulation occurs in most bitches approximately two days after the LH surge. At the time of ovulation, progesterone concentrations rise to between 4 and 10 ng/mL [3,5,7]. The determination of blood progesterone concentration is used for indirect estimation of the LH surge and of the time-point of ovulation in bitches [8,9,10].

A preovulatory rise in progesterone was also observed in humans [11,12] and rats [13]. It is believed that in both these species the LH surge and ovulation are initiated by a preovulatory rise of progesterone [14]. To explain the role of progesterone in the induction of the preovulatory LH surge, the progesterone receptor antagonist mifepristone (RU 486) was used. The administration of this antigestagen significantly reduced the pre-ovulatory LH surge and ovulation in rats [15,16] and women [17]. A similar observation was made in rats using the 3β-hydroxysteroid-dehydrogenase inhibitor trilostane [18]. These studies showed that the preovulatory rise of progesterone is important for the regulation of LH secretion and the ovulatory process in rats and humans.

However, there are only a few studies on the role of preovulatory progesterone for preovulatory LH release [19] and ovulation in the bitch [20,21]. More recently, in order to investigate the role of progesterone, its mimicked withdrawal by the application of the progesterone receptor antagonist aglepristone (RU 534) was described in the dog [19,20,22]. Aglepristone blocks receptor-mediated effects of progesterone, irrespective of its origin [23].

The aim of the study was to evaluate the influence of administration of aglepristone in mid-proestrus on progesterone concentration, LH release, and occurrence of ovulation in the bitch.

## 2. Materials and Methods

### 2.1. Animals and Study Design

The study was approved by the Local Ethics Committee (permit Nr. 44/2013) and performed in accordance with animal protection regulations.

Fourteen clinically healthy adult bitches of mixed breeds, aged 2 to 8 years, and weighing from 8 to 30 kg with normal reproductive history, were included in this study. The bitches were housed in indoor-outdoor runs, fed a complete standard dry diet twice a day, and provided with water ad libitum.

When the bitches entered spontaneous heat and displayed proestrus signs, they were randomly assigned into two groups: experimental group (*n* = 7) and control group (*n* = 7). Experimental bitches were treated with aglepristone (Alizin^®^, Virbac, Carros, France) at the dose of 10 mg/kg body weight s.c. on days 4 and 5 of proestrus when progesterone concentration was below 1 ng/mL and the percentage of superficial cells increased. Control animals received s.c. injections of saline.

The collection of samples for LH and progesterone started on the day of aglepristone administration. For progesterone determination, blood was collected daily from the vena saphena into heparinized tubes until the first day of cytological diestrus. Tubes were centrifuged within 10 min after blood collection and the plasma was stored at +5 °C until assayed the same day. For LH determination, blood was collected daily into heparinized tubes until progesterone concentration was above 1.5 ng/mL, then every 8 h until progesterone concentration was above 5 ng/mL, and again once a day until the first day of cytological diestrus. Tubes were centrifuged within 10 min after blood collection, the plasma was immediately placed at −24 °C and stored until use.

### 2.2. Clinical Observations and Vaginal Cytology

Clinical observations included sexual behavior, vulval swelling, vaginal effluent, and vaginoscopic appearance of the vaginal mucosa. Vaginal smears were stained with Shorr stain and examined microscopically according to standard cytologic criteria for the canine cycle [24].

### 2.3. Hormone Assays

The concentration of progesterone was determined by radioimmunoassay [25]. The sensitivity of the method was 0.06 ng/mL. The LH concentration was measured using of a sandwich-type immune-enzymatic test (LH-Detect^®^ for canines, ReproPharm, Nouzilly, France) [26].

### 2.4. Estimation of Ovulation

Ovulation was identified on the basis of the rise of progesterone concentration above 5 ng/mL and retrospectively by the diestral shift in vaginal cytology, when there was a sudden increase in parabasal cells and neutrophils [27].

### 2.5. Statistical Analysis

Hormone values are shown as the mean ± SE and the length of interval from the first administration of aglepristone to the LH peak as the mean ± SD. To describe the preovulatory LH surge, the area under the curve (AUC) was calculated. To test for differences in hormones concentrations and AUC for LH between the groups, the Student’s *t* test was used (Sigma Plot 6.0 ^®^ Systat Software Inc., San Jose, CA, USA). The level of significance was set at *p* < 0.05.

## 3. Results

### 3.1. Progesterone Plasma Levels

**Figure 1:**
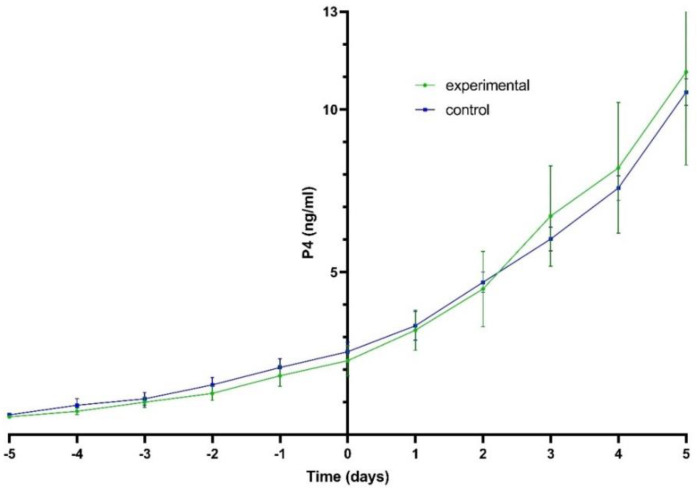
The profiles of P4 (ng/mL, mean ± SE) in peripheral plasma in experimental bitches treated with aglepristone (*n* = 7) and in control bitches (*n* = 7). Hormone profiles were related to the day of the LH peak (Day 0).

The P4 concentration showed a similar pattern in both groups. At the onset of the LH surge, the progesterone concentration was 1.2 ± 0.21 ng/mL in experimental bitches and 1.5 ± 0.23 ng/mL in control bitches (Figure 1). The difference was not statistically significant (*p* > 0.05).

### 3.2. LH Plasma Levels

**Figure 2:**
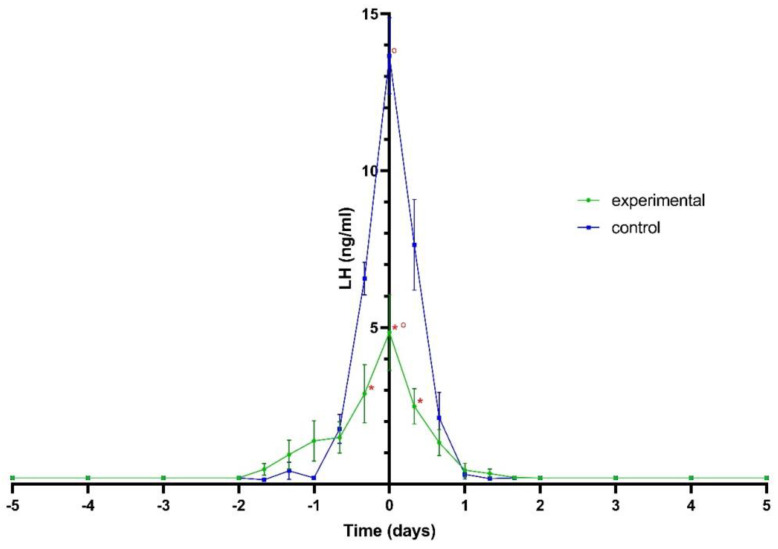
The profiles of LH (ng/mL, mean ± SE) in peripheral plasma in experimental bitches in experimental bitches treated with aglepristone (*n* = 7) and in control bitches (*n* = 7). Hormone profiles were related to the day of LH peak (Day 0).

The peak LH value in bitches treated with aglepristone was significantly lower (*p* < 0.05) than that in control bitches (Figure 2).

### 3.3. AUC for LH

The AUC for LH in experimental bitches was significantly different (*p* < 0.05) from the AUC calculated for the control bitches (Table 1). The interval from first administration of aglepristone to the LH peak was not statistically significant (*p* > 0.05) between experimental and control groups (Table 1). Ovulation occurred in all animals in both groups.

## 4. Discussion

Research on the role of progesterone in the regulation of LH and ovulation under in vivo conditions is difficult. Treatment with aglepristone, which is a known progesterone receptor blocker, allowed us to develop a non-invasive experimental model for temporary elimination of the endogenous progesterone biological action. In the present study, we demonstrated that the withdrawal of progesterone by administration of aglepristone in mid-proestrus significantly reduced the LH surge, but it did not inhibit the LH peak in bitches. In contrast, in the study of Troisi et al. [19], no LH peaks were detected in the treated group. However, the treatment regime was different (two injections of aglepristone 24 h apart in the early follicular phase, the third injection 7 days later) and LH was measured by a different test. Nevertheless, the results of both studies indicate that an increase in progesterone plays an important role in the preovulatory release of LH in the dog. This finding is in line with previous studies showing that the antiprogestin RU 486 reduces LH secretion in proestrus rats [15,16]. Liu and Yen [11] reported that progesterone is essential to establish a normal LH surge in women. Batista et al. [17] showed that administration of the progesterone antagonist RU 486 prevented the LH surge in women. This effect could be reversed by adding small doses of P4 to RU 486 therapy after the emergence of a mature follicle, suggesting that P4 plays an important, if not critical, role in the initiation of the midcycle gonadotropin surge.

Progesterone exerts an important regulating action on the magnitude of the LH surge. The mechanism by which progesterone influences the LH release remains unclear. Concannon [2] suggested that decline in the estradiol to progesterone (E:P) ratio is the ultimate trigger for the LH surge in the bitch. The rise in progesterone before the LH surge onset would participate in facilitating the surge-triggering action of the decline in the E:P ratio. Mahseh and Brann [28] believed that the preovulatory LH surge is regulated by the integrative effects of estradiol and progesterone. The possible sites of action of progesterone in inducing the preovulatory gonadotropin surge could be the anterior pituitary, in which progesterone could alter the response of the pituitary to GnRH, and/or the hypothalamus, where progesterone could influence GnRH release.

Intriguingly, the reduction of the LH surge did not result in anovulation in bitches. Ovulation occurred in all bitches treated with aglepristone. The number of ovulated follicles was not determined. There may be differences in the response of individual follicles to the low LH surge. However, Reynoud et al. (2015) treated bitches with aglepristone at the end of proestrus and performed ovariohysterectomy after ovulation. The mean number of corpora lutea did not differ between treated and control groups. Similarly, Troisi et al. [19] and Reynoud et al. [20] reported that aglepristone administration during late proestrus did not cause any disturbances in the ovulation occurrence. The administration of the progesterone antagonist onapristone before ovulation in ewes also did not affect ovulation [29]. In contrast, studies in rats [15,16] and humans [17,30] showed that mimicked withdrawal of progesterone by administration of the antigestagen RU 486 inhibited ovulation. Thus, the ovulation response on withdrawal of progesterone seems to be species specific. The exact mechanisms of progesterone action in promoting ovulation in the bitch remains to be elucidated. Recently, Dozortsev et al. [14] proposed a new ovulation paradigm in which progesterone rise activates the GnRH signaling pathway, with an ensuing LH surge. On the other hand, it should be noted that ovulation itself is a biological phenomenon regulated by numerous other systemic and local factors.

Progesterone in cyclic bitches is also essential for sexual behavior and oocyte maturation and fertilization. In bitches treated with aglepristone during the early proestrus, the duration of sexual behavior was significantly extended [21]. Aglepristone delayed the resumption of oocyte meiosis and inhibited its progression. In inseminated females, aglepristone prevented sperm progression toward the oviducts and fertilization [20].

In the present study, the administration of aglepristone did not affect the periovulatory progesterone concentration. Similarly, Troisi et al. [19] did not find any differences in progesterone concentration after aglepristone administration during the follicular phase in bitches. Reynoud et al. [20] showed that aglepristone administration at the end of the proestrus and 24 h later caused only temporary lower progesterone concentrations at days 3 and 4 after ovulation in treated bitches compared to that in control bitches. Galac et al. [31] reported that the administration of aglepristone once daily on two consecutive days in a dose of 10 mg/kg body weight, beginning 12 ± 1 days after ovulation, did not influence the length of the luteal phase. However, intervals during which plasma progesterone concentration exceeded 32 nmol/L were significantly shorter in treated bitches than in untreated control dogs (39 ± 2 days vs. 47 ± 5 days). In contrast, administration of aglepristone to non-pregnant bitches in the mid-luteal phase induced early luteal regression [32]. Similar observations have been noted in pregnant bitches and bitches with pyometra treated with aglepristone [21,33,34,35]. Shortening of the luteal phase in pregnant bitches after administration of aglepristone in mid-gestation was associated with increased plasma levels of the metabolite of prostaglandin F_2_α, 15-keto-13,14-dihydro-PGF_2_α (PGFM) [35]. Thus, the effect of aglepristone on progesterone concentration depends on the phase of the reproductive cycle. It may be mediated by suppression of the luteotropic support of pituitary gonadotropins or by inhibition of the positive effects of progesterone acting as an autocrine factor with positive feedback action on steroidogenesis [34].

## 5. Conclusions

The presented study showed that mimicked withdrawal of progesterone by administration of aglepristone in the mid-proestrus significantly reduced the preovulatory LH surge, but it had no effect on periovulatory progesterone concentration or the occurrence of ovulation. This indicates that the preovulatory increase in progesterone plays an important role in the regulation of the LH surge, but its role in triggering ovulation in the bitch seems to be limited.

## Figures and Tables

**Table 1:** The AUC for LH, interval from the first administration of aglepristone to the LH peak, and ovulation rate in experimental and control bitches.

Variables	Experimental Group (*n* = −7)	Control Group (*n* = 7)
The AUC for LH (ng/mL/d, mean ± SE)	6.85 ± 1.21 ^a^	12.25 ± 1.35 ^b^
Interval from first administration of aglepristone to the LH peak (days, mean ± SD)	4.25 ± 1.26	5.36 ± 1.45
Ovulation rate (%)	100.00	100.00

^a,b^—difference is statistically significant at *p* < 0.05.
